# A record-linkage study of the development of hepatocellular carcinoma in persons with hepatitis C infection in Scotland

**DOI:** 10.1038/sj.bjc.6604563

**Published:** 2008-08-26

**Authors:** S A McDonald, S J Hutchinson, S M Bird, C Robertson, P R Mills, J F Dillon, D J Goldberg

**Affiliations:** 1 Health Protection Scotland, Clifton House, Clifton Place, Glasgow G3 7LN, UK; 2 Department of Statistics and Modelling Science, University of Strathclyde, Richmond Street, Glasgow G1 1XH, UK; 3 MRC Biostatistics Unit, Institute of Public Health, University Forvie Site, Robinson Way, Cambridge CB2 2SR, UK; 4 Gartnavel General Hospital, 1053 Great Western Road, Glasgow G12 0YN, UK; 5 Ninewells Hospital and Medical School, Dundee DD1 9SY, UK

**Keywords:** hepatocellular carcinoma, hepatitis C, record-linkage study, alcohol

## Abstract

We investigated trends in first-time hospital admissions and deaths attributable to hepatocellular carcinoma (HCC) in a large population-based cohort of 22 073 individuals diagnosed with hepatitis C viral (HCV) infection through laboratory testing in Scotland in 1991–2006. We identified new cases of HCC through record-linkage to the national inpatient hospital discharge database and deaths registry. A total of 172 persons diagnosed with HCV were admitted to hospital or died with first-time mention of HCC. Hepatocellular carcinoma incidence increased between 1996 and 2006 (average annual change of 6.1, 95% confidence interval (CI): 0.9–11.6%, *P*=0.021). The adjusted relative risk of HCC was greater for males (hazard ratio=2.7, 95% CI: 1.7–4.2), for those aged 60 years or older (hazard ratio=2.7, 95% CI: 1.9–4.1) compared with 50–59 years, and for those with a previous alcohol-related hospital admission (hazard ratio=2.5, 95% CI: 1.7–3.7). The risk of individuals diagnosed with HCV developing HCC was greatly increased compared with the general Scottish population (standardised incidence ratio=127, 95% CI: 102–156). Owing to the advancing age of the Scottish HCV-diagnosed population, the annual number of HCC cases is projected to increase, with a consequent increasing burden on the public healthcare system.

The aetiology of hepatocellular carcinoma (HCC) includes excessive alcohol consumption and chronic infection with the hepatitis B or C virus. The incidence of HCC has increased in developed countries worldwide (Deuffic *et al*, 1999; El-Serag and Mason, 1999; Law *et al*, 2000), but data are limited on its cause. However, modelling initiatives have projected increasing numbers of HCC deaths attributable to hepatitis B and/or hepatitis C virus (HCV) infection (Deuffic-Burban *et al*, 2004, 2007; Sweeting *et al*, 2007).

In Scotland, HCV is responsible for a significant burden on healthcare (Hutchinson *et al*, 2006). Meeting the public health challenge of providing sufficient resources for treatment and care of patients with HCV requires up-to-date data on morbidity and mortality. However, national surveillance systems for detecting advanced HCV-related liver disease are currently lacking in the United Kingdom and elsewhere. The existence of high-quality national HCV diagnosis and hospital discharge databases provided the opportunity to use record-linkage techniques to investigate the annual number of new diagnoses of, and deaths from, HCV-related HCC.

Our principal goal was to investigate trends in the annual incidence of HCC in the entire HCV-diagnosed population in Scotland through record linkage to hospital admission and deaths records. We also aimed to confirm previously reported associations between risk factors and progression to HCC. Up-to-date information regarding factors affecting the rate of development of HCC in individuals diagnosed with HCV is needed for informing clinical management and care, and for refining guidelines for early detection.

## Materials and methods

The design was an observational record-linkage study involving national HCV diagnosis and hospital discharge databases and the national deaths and cancer registries, and subsequent analyses of the incidence of HCC among a retrospective cohort of all persons diagnosed with HCV infection. HCV-related HCC incidence was calculated using hospital discharge data, as this was available until 2006, and validated against cancer registrations (available until 2004).

Health Protection Scotland (HPS) maintains a database of all persons who have been diagnosed as HCV positive in Scotland since testing commenced in 1991 (Shaw *et al*, 2003); laboratory detection of hepatitis C antibody is a requirement for inclusion. This database contains the following non-named information: surname soundex code, forename initial, date of birth, sex, and the first part of the postcode of residence, as well as data concerning risk activities. As of 31 December 2006, the database contained records for 22 073 persons diagnosed as HCV positive (HPS, 2007), who comprise the study population.

The Scottish Morbidity Records (SMR01) is an episode-based patient record held by Information Services Division (ISD) of all general acute inpatient and day case hospital discharges. Discharge diagnoses use International Classification of Diseases (ICD) Ninth Revision for discharges from 1989 to 1995, and Tenth Revision for discharges from 1996 to 2006. The Scottish Morbidity Cancer Records (SMR06) is a national database containing all cancer notifications in Scotland; data include date of registration and cancer site (but not aetiology) and were available up to the end of 2004. ISD routinely combines the SMR01 and SMR06 data with death registrations held by the General Register Office for Scotland to form a linked data set.

Linkage of records between the HCV diagnosis database and the previously linked SMR01/SMR06/deaths registry was carried out by ISD using probabilistic record-linkage techniques (Kendrick and Clarke, 1993). ISD had previously estimated the error rate (either false positives or false negatives) of their procedure to be less than 5% (Kendrick and Clarke, 1993). The resulting linked data set was anonymised before transfer to HPS for analysis. Linkages were approved by the Privacy Advisory Committee, which advises on confidentiality issues involving data held on NHS Scotland patients.

The main outcome of interest was the annual numbers of first hospital admissions (or death, if no previous admission) with mention of HCC. Combining admission and death information to estimate the annual incidence of HCC is justified because of the relatively short lag (see Results) between first HCC admission and death. Diagnosis/cause-of-death codes indicated the development of HCC; specifically, ICD-10 C22.0 or ICD-9 155.0 mentioned as either a primary or secondary discharge diagnosis in a linked SMR01 hospitalisation record, or as either the underlying or a contributing cause of death in a linked death record. The terms admission and discharge are used interchangeably; it is assumed that discharge diagnosis codes encode the reason(s) for admission.

### Data analysis

No probabilistic linkages to the linked SMR01/06/death records were achieved if the HCV record lacked date of birth and two or more other identifiers. By these criteria, records for 1324 out of 22 073 persons had insufficient identifiers and thus were excluded from further analyses.

We carried out two types of analyses. First, annual trends in the numbers of persons diagnosed with HCV developing HCC were estimated using Poisson regression. We analysed annual trends in counts rather than rates because the expansion of the HCV diagnosis database since its inception – with increasing proportions of persons at earlier stages of disease progression being identified and added – means that rates calculated for recent years would reflect more outcome-free person-years of follow-up than rates for earlier years. The eligible population was restricted to those with an HCV diagnosis date before or within 1 year after their first hospital admission for HCC. Data for individuals for whom first mention of HCC occurred more than 1 year before their HCV diagnosis date were excluded (*n*=6), as were individuals who died from any cause more than 1 year before HCV diagnosis (*n*=23). Of the 20 749 persons diagnosed with HCV with sufficient identifiers for record linkage, 20 720 met this 1-year constraint. Analysis of annual counts was restricted to the period 1996–2006, owing to limited HCV antibody testing before 1996. We additionally compared the annual incidence of diagnosed HCV-related HCC derived from the hospital discharge/death records linkage with the corresponding annual numbers of linked SMR06 cancer registrations over the period 1996–2004.

Second, an analysis of time from HCV diagnosis to first admission/death with mention of HCC was undertaken. The observation period was defined to start 14 days after HCV diagnosis; thus records for persons diagnosed within the 14-day period before 31 December 2006 (*n*=44) and persons first hospitalised for HCC or who died of any cause before HCV diagnosis (*n*=328), or within 14 days following this date (*n*=154), were excluded. Of the excluded records, 28 were first diagnosed as HCV positive subsequent to their first hospital admission for HCC and 300 were first diagnosed after death. Nine individuals were first hospitalised with HCC, 3 died with mention of HCC, and 142 died of other causes within 14 days of HCV diagnosis. Confining analysis to persons who are outcome free at the start of follow-up reduces bias due to an increased risk of hospitalisation at the time of diagnosis for patients presenting with established disease. Time at risk (in person-years) was then calculated from the start of the observation period to the earliest of first hospital admission for HCC, date of death or the end of the observation period (31 December 2006). Records for 20 223 individuals diagnosed with HCV were eligible for this analysis.

We used Cox proportional hazards regression analysis to assess the strength of association between risk factors (given below) and the time to first hospital admission/death with mention of HCC. Age and whether the person had a previous admission for an alcohol-related condition were treated as time-dependent covariates; sex was included as a time-independent covariate. Previous admission with mention of hepatitis B virus (HBV) infection was not included in the multifactorial analysis, as it was unassociated with the risk of developing HCC. Human immunodeficiency virus (HIV)-co-infected status was also considered as a covariate, but record linkage to a national HIV diagnosis database indicated no HCC cases to be HIV-co-infected (McDonald *et al*, in press). Kaplan–Meier methods were used to estimate the age-dependent probability of developing HCC, according to sex, risk activity leading to infection, and whether or not the person had a previous admission with mention of cirrhosis or an alcohol-related condition.

We computed standardised incidence ratios (SIRs) for HCC in the HCV-diagnosed population by age, sex, and calendar-year standardising to expected incidence rates derived from all HCC registrations in Scotland during 1996–2004 and the national mid-year population estimates for the same period (ISD, 2008). All data analyses were carried out using R version 2.4.0 (R Foundation for Statistical Computing, 2006).

For each person diagnosed with HCV, the occurrence of at least one alcohol-related hospital episode was coded as a time-dependent variable. This involved searching the linked hospital records for alcohol-related discharges occurring before the date of first mention of HCC, or, for those with no HCC admission/death, at any time before 31 December 2006. The set of alcohol-related diagnosis codes comprised alcohol use (ICD-10 Z72.1), mental and behavioural disorders due to use of alcohol (ICD-10 F10; ICD-9: 291, 303, 305), degeneration of nervous system due to alcohol (ICD-10 G31.2, G62.1, G72.1, I42.6, K29.2; ICD-9 357.5, 425.5, 535.3), toxic effects of alcohol (ICD-10 T51.0, T51.9; ICD-9 980.0), alcoholic liver disease (ICD-10 K70.1-3; ICD-9 571.0–571.2), alcohol-induced chronic pancreatitis (ICD-10 K86.0), evidence of alcohol involvement (ICD-10 Y90-1), finding of alcohol in blood (ICD-10 R78.0; ICD-9 790.3), alcohol rehabilitation (ICD-10 Z50.2), personal history of psychoactive substance abuse (ICD-10 Z86.4; captures non-current mental/behavioural disorders due to use of alcohol), and accidental or intentional self-poisoning by and exposure to alcohol (ICD-10 X45, X65; ICD-9 E860.0, E860.9).

Additional risk factors identified by previous research as predictors of the development of HCC were coded for each person. These included age (time-dependent: <50, 50–59 and 60+ years) and reported risk activity leading to infection (injecting drug use (IDU), non-IDU, not known). The non-IDU group included persons who had reported potential acquisition of HCV infection through receipt of blood or blood products, tattoo/body piercing, needlestick injury, sexual contact, or perinatal transmission. Previous hospitalisation for cirrhosis was also assessed by searching the database linkage for mention of non-biliary cirrhosis among the discharges occurring before first mention of HCC, or at any time before 31 December 2006 for those with no admission/death for HCC. Cirrhosis codes comprised alcoholic cirrhosis of liver (ICD-10 K70.3; ICD-9 571.2), cirrhosis of liver without mention of alcohol (ICD-9 571.5), and other and unspecified cirrhosis of liver (ICD-10 K74.6). The occurrence of a previous hospital discharge with mention of HBV (ICD-10 B16, B18.0, B18.1; ICD-9 070.2, 070.3) was similarly noted.

## Results

The majority (68%) of the study population were male (Table 1); at the time of HCV diagnosis, 42% were under the age of 30 years and 13% were 45 years or older (median=32 years). A total of 31% had a previous hospital discharge related to alcohol, 3.5% for cirrhosis, and 5.6% with mention of hepatitis B.

Over a median follow-up of 5.6 years (range=0–22.0) for all 20 720 persons diagnosed with HCV, 172 were either admitted with first-time mention of HCC (*n*=145) or died without being previously hospitalised with HCC (*n*=27); that is, their first and only indication of HCC was recorded on the death record. The median time from HCV diagnosis date to first-time mention of HCC was 2.3 years (0.2, 1.7, and 3.5 years, for HCC calendar periods <1996, 1996–2000, and >2000, respectively). Of the 145 individuals first hospitalised with HCC, 115 subsequently died, of whom 95 had their underlying or a contributing cause of death recorded as HCC; 55% (11 out of 20) of the other deaths specified a liver-related underlying cause. Twenty-two out of 172 individuals were diagnosed with HCV following their first HCC admission/death; the majority of these diagnoses were made within 2 weeks (*n*=19) of first HCC admission/death.

For those individuals who died with an underlying cause of HCC (tumour) subsequent to their first hospitalisation for HCC (*n*=83), the median lag between first-time admission and death was 81 days. Forty-four per cent (76 out of 172) of HCC cases had a previous admission for cirrhosis, and 31% (53 out of 172) had a previous alcohol-related hospitalisation. The median lag between first admission for cirrhosis and first-time mention of HCC was 2.3 years. Among only those persons hospitalised for cirrhosis, the one- and two-year cumulative risks of developing HCC following first admission for cirrhosis were 3.5 and 6%, respectively.

Hepatitis C virus was listed as a discharge diagnosis in 39% (56 out of 145) of HCC admissions, and as the underlying or a contributing cause in 41% (11 out of 27) of deaths with first-time mention of HCC.

Table 2 shows the number of first-time hospital admissions/deaths per year over the period 1996–2006, stratified by age group at admission/death. There was a significant increasing trend in the overall number of first-time admissions/deaths (average annual change=6.1%, 95% CI: 0.9–11.6%, *P*=0.021). Incorporation of a year by age-group interaction term indicated a significantly greater mean annual change in the 50–59 years age group (16.3%, 95% CI: 6.1–27.5%, *P*=0.028) compared with the <50 years age group.

The incidence of HCC in the HCV-diagnosed population as determined from hospital discharge/death records was compared with the linked (HCV-related) SMR06 cancer registrations over the period 1996–2004. There was good agreement between the annual numbers of first-time HCC admissions/deaths and the SMR06 data. In the study period, there were 108 new entries for HCC in the cancer registry (12, 16, 4, 11, 6, 13, 15, 14, and 17 registrations for the years 1996 through 2004, respectively), 7 fewer than the 115 new cases of HCC determined from the hospital admission/death record linkage in the same period. Three of the cases absent from the linked cancer registrations were deaths without previous admission for HCC. Five of the 108 cancer registrations were not found in the hospital admission/death record linkage.

**Figure 1:**
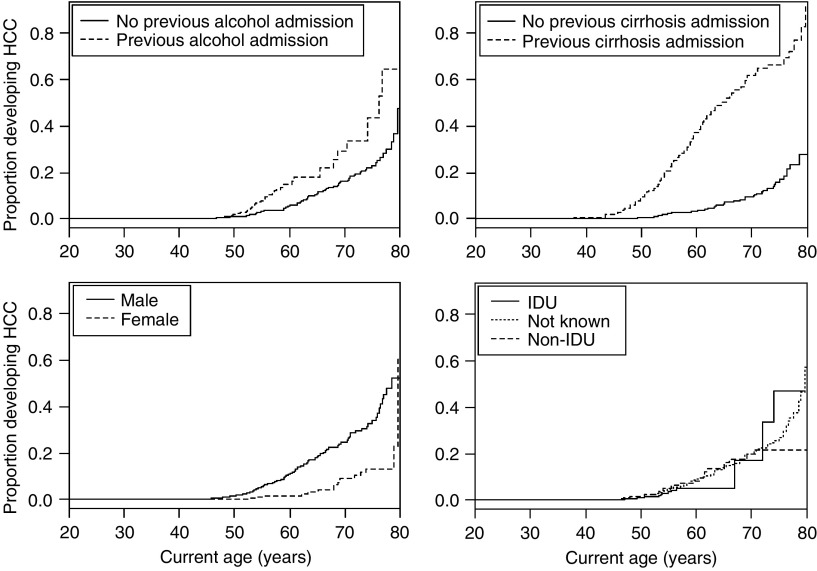
Proportion of individuals who were hospitalised or died with first-time mention of HCC over the period 1 January 1991 to 31 December 2006 as a function of (**A**) previous alcohol-related admission; (**B**) previous admission for cirrhosis; (**C**) sex; and (**D**) risk group.

Figure 1 shows the age-dependent proportion of persons with a first-time hospitalisation/death with mention of HCC as a function of sex and whether or not the person had a previous alcohol- or cirrhosis-related admission. There was no difference in the unadjusted cumulative probability of developing HCC according to previous alcohol-related admission status (log-rank test, *P*=0.41). In contrast, a previous admission for cirrhosis was associated with a much greater proportion of persons developing HCC (*P*<0.001).

Table 3 shows adjusted hazard ratios derived from multifactorial Cox proportional hazards regression analysis, with sex, current age (<50, 50–50, and 60+ years), and previous alcohol-related admission as covariates. Violation of the proportional hazards assumption was assessed graphically and tested using Schoenfield residuals (global *P*=0.63). Males were more likely than females to develop HCC (hazard ratio=2.7, 95% CI: 1.7–4.2). Compared with the reference age group (50–59 years), older age (60+ years) was associated with an increased risk of first-time admission/death (HR=2.7, 1.9–4.1) and younger age (<50 years) with a decreased risk (HR=0.03, 95% CI: 0.02–0.05); there was a 2.5-fold increased risk associated with a previous alcohol-related admission (HR=2.5, 95% CI: 1.7–3.7).

Over the period 1996–2004, a total of 1304 HCC cases were registered in Scotland (ISD, 2008). The estimated prevalence of diagnosed HCV infection among all HCC cases was therefore 8.8% (115 out of 1304; 1996–1999 only: 8.4% (45 out of 535); 2000–2004: 9.1% (70 out of 769)). The SIR for HCC was 127 (95% CI: 102–156). We also calculated separate SIRs for males and females: 118 (95% CI: 93–149) and 179 (95% CI: 106–283), respectively.

Over the entire observation period (1991–2006), 108 HCV-diagnosed persons died with an underlying cause of death recorded as HCC, representing 4% of all deaths (*n*=2622) that occurred during this period. One- and two-year probabilities of death with mention of HCC subsequent to first-time hospitalisation for HCC were 53 and 58%, respectively; the respective one- and two-year probabilities of death from any cause were 62 and 70%. Of those who died of HCC, 39% (42 out of 108) had a previous admission for cirrhosis, and 32% (35 out of 108) had a previous alcohol-related admission.

## Discussion

In this study, we generated up-to-date estimates of the annual incidence of HCC in the Scottish HCV-diagnosed population and assessed the relative risk of developing HCC associated with various variables. A key strength of this study is the use of national data sources, which provided over 120 000 person-years of follow-up.

The incidence of HCC increased over the period 1996–2006. Consistent with this trend, there was an increasing trend in the number of HCV-related deaths from liver cancer in Scotland over the period 1996–2005 (Palmateer *et al*, 2007). Similar rising trends in the incidence of HCV-related HCC have been observed over overlapping periods in England (1996–2004; Sweeting *et al*, 2007) and Australia (1990–2002; Amin *et al*, 2007).

Of those persons who died subsequent to their first hospitalisation with HCC, only 83% (95 out of 115) had HCC mentioned in their death record, suggesting that studies that rely on death registry data may underestimate HCC incidence. This is particularly relevant for modelling studies using back-calculation methods to estimate the past incidence of chronic HCV infection (Deuffic-Burban *et al*, 2004, 2007; Sweeting *et al*, 2007). Our finding that only 40% of hospitalisations and 38% of deaths with first-time mention of HCC had an HCV code listed also highlights the limitations of registry methods.

In the general Scottish population, the overall incidence rate for HCC in the period 1996–2004 was 2.9 per 100 000 persons (ISD, 2008). Incidence was many times greater in the HCV-diagnosed population; over the period 1996–2004, we obtained an SIR of 127, five times larger than the SIR of 27 (95% CI: 23–31) in a New South Wales (NSW), Australia, study (Amin *et al*, 2006; revised SIR from J Amin, personal communication). This large discrepancy in SIRs is attributable to differences in the definition of time at risk; in the NSW study, the observation period is defined to start 1 year after HCV diagnosis, as opposed to 14 days in this study. Notably, the estimated prevalence of diagnosed HCV among all HCC cases (8.8%) in Scotland was lower than the value (13.7%) reported for NSW (Amin *et al*, 2007).

A shift in the incidence of HCC in one age group (40–60 years) in a US population-based study has been reported (El-Serag and Mason, 1999), reflecting the delay between disease onset and HCV infection (thought to occur in the 1960s and 1970s). We observed the largest average annual change in HCC incidence (16.3%) in the 50–59 years group; for these persons, the mean age at diagnosis was 52, suggesting that infection occurred 20–25 years earlier, in the 1970s.

Time-to-event analysis indicated that male sex, older age (50+ years), and the occurrence of a previous hospital discharge for an alcohol-related condition were all factors that greatly increased the relative risk of developing HCC. These results concur with previous research (Niederau *et al*, 1998; Schafer and Sorrell, 1999; Kuper *et al*, 2001).

Cirrhosis is an important risk factor for HCC, with HCC developing in cirrhotic livers (irrespective of viral hepatitis infection) with an annual incidence of 3% (Colombo *et al*, 1991; Velazquez *et al*, 2003). An estimated 5–15% of chronic HCV-infected individuals develop cirrhosis within 20 years after diagnosis (Freeman *et al*, 2001; Seeff, 2002; Benvegnù *et al*, 2004). In HCV-infected patients with established cirrhosis, the annual incidence and 5-year probability of HCC developing have been estimated at 3.5% (Hutchinson *et al*, 2005) and 28–30% (Benvegnù *et al*, 2004; Sola *et al*, 2006), respectively.

A previous admission with mention of cirrhosis was recorded for 45% of HCC cases and was followed by hospital admission/death with first-time mention of HCC at a median lag of 2.3 years. Given the relatively young age distribution of the Scottish HCV-diagnosed population (median age at diagnosis is 32) and the relatively short observation time, the number of persons developing cirrhosis will be expected to increase as this population ages. We expect to see a corresponding increase in the number of individuals diagnosed with HCV infection who progress to HCC.

Previous hospital admission for an alcohol-related condition was associated with a 2.6-fold increased risk of developing HCC. Problem alcohol use, to the extent that it is captured by our proxy variable, appear to be highly prevalent in our study population; 31% of the cohort had been previously admitted to hospital with an alcohol-related diagnosis.

High levels of alcohol consumption, particularly above 350 g per week, have been associated with accelerated progression to cirrhosis in chronic HCV-infected persons (Ostapowicz *et al*, 1998; Freeman *et al*, 2003; Monto *et al*, 2004). There is less evidence that alcohol increases the risk of developing HCC once cirrhosis is established (Adami *et al*, 1992; Teli *et al*, 1995; Velazquez *et al*, 2003; Planas *et al*, 2004; also see Miyakawa *et al*, 1996; Tsutsumi *et al*, 1996), suggesting an indirect mechanism by which alcohol increases the risk of HCC (Kuper *et al*, 2001). Given the strong association between excessive alcohol intake and cirrhosis, one measure for reducing the risk of HCC in the HCV-infected population is to limit alcohol intake.

Development of HCC in HCV-infected persons usually requires 15–25 years (Schafer and Sorrell, 1999; Freeman *et al*, 2001); the median lag between HCV diagnosis and hospitalisation/death with first mention of HCC in our study was much shorter, 2.3 years. Our study population, in particular those aged 40 and over at diagnosis, were likely tested and diagnosed as HCV antibody positive many years after infection, and in numerous cases, diagnosis was concurrent to or shortly followed first hospital admission with HCC. Given accurate data on the date of infection, we would expect the time to HCC development to be more consistent with previous studies.

The incidence of HCV-related HCC may have been underestimated due to (i) insufficient identifiers in the HCV Diagnosis database for record linkage and (ii) an unknown, but likely small, number of HCC cases that were not tested (and so never diagnosed) for HCV infection during 1996–2006.

In conclusion, the significance of excess risk of HCC and the trend for increasing annual numbers of 50- to 59-year-olds developing HCC are consistent with ageing of the members of the Scottish HCV-diagnosed population and the consequent progression to severe liver disease. Our study has highlighted the growing burden on public healthcare resources from HCV-related outcomes such as HCC.

## Figures and Tables

**Table 1:** Characteristics of persons in the national HCV diagnosis database whose records contained sufficient identifiers for record-linkage, and the HCV diagnosis date was no more than 1 year following the first mention of HCC, if any (data up to 31 December 2006; *N*=20 720)

**Variable**	**Group**	***N***	**(%)**
Sex	Male	14 082	(68.0)
	Female	6597	(31.8)
	Not known	41	(0.2)
Age at HCV diagnosis (years)	<30	8673	(41.8)
	30–44	9320	(45.0)
	45+	4726	(13.2)
Period of HCV diagnosis	Pre-1996	3336	(16.1)
	1996–2000	7939	(38.5)
	2001–2006	9445	(45.6)
Risk group	IDU	12 560	(60.6)
	Non-IDU	1581	(7.6)
	Not known	6579	(31.8)
Previous admission, alcohol-related	Yes	6464	(31.2)
	No	14 256	(68.8)
Previous admission with cirrhosis	Yes	734	(3.5)
	No	19 986	(96.5)
Previous admission with hepatitis B	Yes	1166	(5.6)
	No	19 554	(94.4)

HCV=hepatitis C virus; IDU=current/former injecting drug user. An alcohol-related admission is defined as hospitalisation with a discharge diagnosis code of at least one of the following: (ICD-10) Z72.1, F10, G31.2, G62.1, G72.1, I42.6, K29.2, T51.0, T51.9, K70.1-3, K86.0, Y90-1, R78.0, Z50.2, Z86.4, X45, X65, (ICD-9) 291, 303, 305, 357.5, 425.5, 535.3, 571.0-571.2, 790.3, E860.0, E860.9. Admission with cirrhosis is defined similarly as follows: (ICD-10) K70.3, K74.6, (ICD-9) 571.2, 571.5, and admission with hepatitis B is defined similarly as follows: (ICD-10) B16, B18.0, B18.1, (ICD-9) 070.2, 070.3.

**Table 2:** Number of first-time hospital admissions/deaths with mention of HCV-related HCC over the period 1996–2006, by age at admission, sex, risk group, and previous admission for cirrhosis

	**Year of admission/death**											**Mean annual change**		
**Level**	**1996**	**1997**	**1998**	**1999**	**2000**	**2001**	**2002**	**2003**	**2004**	**2005**	**2006**	**%**	**(95% CI)**	***P***
*Age at admission/death*														
<50	5	5	1	2	1	1	5	2	3	3	4	−0.9	(−12.2, 10.5)	0.87
50–59	2	4	3	2	1	9	3	2	7	8	11	16.3	(6.1, 27.5)	0.001
60+	5	9	1	6	8	6	8	8	6	9	5	2.7	(−4.6, 10.6)	0.48
All	12	18	5	10	10	16	16	12	16	20	20	6.1	(0.9, 11.6)	0.021
														
*Sex*														
Male	11	14	4	8	9	14	14	7	14	17	18	6.4	(0.7, 12.4)	0.027
Female	4	5	1	2	1	2	2	5	2	3	2	4.5	(−7.7, 18.4)	0.49
														
*Risk group*														
IDU	2	4	0	2	0	2	1	1	6	1	5	8.8	(−4.4, 23.8)	0.20
Non-IDU	2	3	1	2	2	1	3	3	3	5	1	4.7	(−7.3, 18.4)	0.45
Not known	8	11	4	6	8	13	12	8	7	14	14	5.8	(−4.5, 12.5)	0.70
														
*Cirrhosis*														
No	4	10	2	7	8	9	7	8	9	12	9	6.7	(−0.3, 14.3)	0.060
Yes	8	8	3	3	2	7	9	4	7	8	11	5.3	(−2.3, 13.5)	0.18
														
*Alcohol*														
No	6	15	4	7	8	11	10	9	8	16	12	5.1	(−1.1, 11.6)	0.11
Yes	6	3	1	3	2	5	6	3	8	4	8	8.4	(−1.0, 18.6)	0.080

IDU=injecting drug user. Presence of cirrhosis is ‘yes’ if patient had a previous hospital admission with mention of cirrhosis. Alcohol is ‘yes’ if patient had a previous hospital admission with mention of alcohol.

**Table 3:** Multifactorial Cox proportional hazards regression results; outcome is first hospitalisation/death with mention of HCC. Analysis is based on data from the entire observation period (1 January 1991 to 31 December 2006)

**Factor**	**Level**	N	**Person-years**	**Rate**	**HR**	**95% CI**	***P*-value**
Sex	Female	23	39 200	0.6	—		
	Male	115	82 202	1.4	2.70	1.72, 4.23	<0.0001
							
Current age group	<50	25	109 952	0.2	0.032	0.020, 0.053	<0.0001
	50-59	48	7481	6.4	—		
	60+	65	4207	15.5	2.75	1.86, 4.06	<0.0001
							
Alcohol-related hospitalisation	None	86	84 339	1.0	—		
	Yes	52	37 334	1.4	2.50	1.70, 3.66	<0.0001

CI=confidence interval; HR=hazard ratio (relative risk); *N*=number of first-time admissions. Rate is per 1000 person-years. Current age group is a time-dependent variable. Alcohol-related hospitalisation is also a time-dependent covariate, changing status from ‘None’ to ‘Yes’ on the date of the first alcohol-related admission.
